# Simultaneous fMRI and tDCS for Enhancing Training of Flight Tasks

**DOI:** 10.3390/brainsci13071024

**Published:** 2023-07-03

**Authors:** Jesse A. Mark, Hasan Ayaz, Daniel E. Callan

**Affiliations:** 1 School of Biomedical Engineering, Science and Health Systems, Drexel University, Philadelphia, PA 19104, USA; 2 Department of Psychological and Brain Sciences, College of Arts and Sciences, Drexel University, Philadelphia, PA 19104, USA; 3 Drexel Solutions Institute, Drexel University, Philadelphia, PA 19104, USA; 4 A.J. Drexel Autism Institute, Drexel University, Philadelphia, PA 19104, USA; 5 Department of Family and Community Health, University of Pennsylvania, Philadelphia, PA 19104, USA; 6 Center for Injury Research and Prevention, Children’s Hospital of Philadelphia, Philadelphia, PA 19104, USA; 7 Brain Information Communication Research Laboratory, Advanced Telecommunications Research Institute International, Kyoto 619-0288, Japan

**Keywords:** aviation, training, neuroergonomics, fMRI, HD-tDCS, brain connectivity, dorsolateral prefrontal cortex, basal ganglia, cerebellum, neurostimulation

## Abstract

There is a gap in our understanding of how best to apply transcranial direct-current stimulation (tDCS) to enhance learning in complex, realistic, and multifocus tasks such as aviation. Our goal is to assess the effects of tDCS and feedback training on task performance, brain activity, and connectivity using functional magnetic resonance imaging (fMRI). Experienced glider pilots were recruited to perform a one-day, three-run flight-simulator task involving varying difficulty conditions and a secondary auditory task, mimicking real flight requirements. The stimulation group (versus sham) received 1.5 mA high-definition HD-tDCS to the right dorsolateral prefrontal cortex (DLPFC) for 30 min during the training. Whole-brain fMRI was collected before, during, and after stimulation. Active stimulation improved piloting performance both during and post-training, particularly in novice pilots. The fMRI revealed a number of tDCS-induced effects on brain activation, including an increase in the left cerebellum and bilateral basal ganglia for the most difficult conditions, an increase in DLPFC activation and connectivity to the cerebellum during stimulation, and an inhibition in the secondary task-related auditory cortex and Broca’s area. Here, we show that stimulation increases activity and connectivity in flight-related brain areas, particularly in novices, and increases the brain’s ability to focus on flying and ignore distractors. These findings can guide applied neurostimulation in real pilot training to enhance skill acquisition and can be applied widely in other complex perceptual-motor real-world tasks.

## 1. Introduction

The training of professionals in complex, high-stress, high-risk fields, such as aviation, is critically important to ensure the safety and wellbeing of both the operator and those whose lives rely on their performance. Modern training methods to produce experts take years of careful instruction and practice to develop pilots with the ability to adapt to different aircraft, weather conditions, and runways and the same can be seen in other such fields as well. Currently, the Federal Aviation Administration requires pilots to have a minimum of 40 h flying before applying for a private license but this number increases to 1500 h when it comes to becoming a commercial pilot [1]. As another example, surgeons must attend four years of medical school and a minimum of five years of residency to be properly trained to perform a variety of life-saving operations and be able to deal with unexpected complications [2]. Unfortunately, these strict but necessary requirements have led to shortages of both pilots and physicians, among other professions [3,4]. This heavy investment of resources into each professional must also keep progressing alongside improvements in technology and training [5]. As such, advances in neuroscience and neuroengineering methods present new opportunities for further enhancement by incorporating functional brain changes that accompany training in increasingly complex and real-world tasks consistent with neuroergonomics [6,7,8,9,10]. Decades of functional neuroimaging studies with diverse modalities suggest learning is associated with functional brain changes [9,11,12,13,14,15]. In addition, neurostimulation techniques have been utilized for improving attention, perception, memory, and other forms of cognition in healthy individuals, leading to better training performance [10,16,17]. Thus, the goal of our study is to combine neuroimaging and neuromodulation during targeted tasks to develop a novel training method to lessen the burden of cultivating new experts in the field.

In this study, we apply transcranial direct-current stimulation (tDCS) to attempt to improve the performance of pilots in a flight simulator and study the resulting brain activity using functional magnetic resonance imaging (fMRI) with continuous measurements before, during, and after training. TDCS is a lightweight and portable, yet highly effective, neuromodulation modality that affects neural plasticity [18] by polarizing neural tissue to enhance long-term potentiation for the faster formation of synaptic connections or otherwise inhibits activity through long-term depression [19,20,21]. It can be used for research and operation everywhere from a lab bench to simultaneously with fMRI, to a plane in flight, making it ideal for neuroergonomic applications [22,23] and enhancing human performance and skill acquisition [24]. Previous research has found benefits from tDCS stimulation of primarily the prefrontal and parietal cortex in working memory training [25] and improvements to visual search associated with fMRI resting state measurements [26] as well as task-related brain activity [17], working memory performance increases and brain activation changes measured with functional near-infrared spectroscopy (fNIRS) [27], an increase in cognitive flexibility associated with a decrease in fNIRS activation [28], differences in performance and the learning rate of N-back and working memory tasks with feedback as well as frontal theta power and brain activation measured with EEG and fNIRS [29], and a higher learning rate of an adaptive difficulty working memory task that found more improvements on less experienced participants [30]. A meta-analysis has shown that tDCS over the DLPFC improves performance on cognitive tasks [31]. Particularly, stimulation to the dorsolateral prefrontal cortex (DLPFC) has been demonstrated to improve cognitive–motor dual-task performance and related EEG prefrontal measures [32], and lowers the dual-task cost of multitasking fine motor control both during and after stimulation [33], and specifically to the right DLPFC. tDCS can improve performance in the Stroop task [34], spatial working memory [35], a complex mathematical working memory task [36], and real-world shooting training, particularly for unskilled learners [37]. The safety of tDCS in healthy populations is well established in the literature [38,39,40,41,42] and, recently, the conservatively considered safe guidelines are being extended to even higher currents [43]. Even amongst the rich literature, very few studies have examined the effects of tDCS on pilots in flight simulators, and to the best of our knowledge, none have been used in real flight experiments to date, augmenting the novelty of our experiment.

Training has a differential effect depending on the baseline performance level of learners. For example, the same practice-based training results in the greatest rate of performance improvement at earlier stages when participants are at lower performance levels and, conversely, improvement approaches a plateau at more advanced levels, resulting in the familiar learning curve [44]. It has also been found that baseline performance level results in differential effects of tDCS—in general, novice operators receive greater benefits, particularly when performance feedback is included in the loop. In visual search tasks [17], associative memory tasks [45], and e-game scenarios [46], learners with initially lower performance levels showed greater improvements due to targeted tDCS stimulation than those with higher starting performance, as well as those in the sham group. Expertise development also results in functional changes in the brain which can be measured with neuroimaging such as fNIRS during task practice, resulting in decreases in activation in task-related brain areas due to experience [47], increases in workload on standardized tasks due to aging [48], and, over a longitudinal training protocol, prefrontal increases and decreases in oxygenation following a learning curve while training on a difficult piloting task [49]. FMRI has also been used to analyze expertise-related brain activity. For example, the motor imagery-based brain activity of novice, expert, and elite archers was studied to determine changes in the brain with mastery [50,51]. The authors outlined four ways in which the brain changes over time: an increase in neural efficiency, the cortical expansion of task-related areas, an increase in specialized (and localized) processing, and subconscious development of internal models. Using neuroimaging techniques to analyze and track these changes over time can allow researchers to further improve neuroadaptive training and enhance the learning process and, in recent years, concurrent use of tDCS and fMRI has even become possible [52]. 

Flight simulators are an ideal medium for studying the effects of tDCS on performance and brain activation, in part because they have a high-performance ceiling and have room to improve for both novice and advanced pilots. They have been used in research involving mental workload, training, and learning, and have been widely utilized for functional neuroimaging experiments to study the effects of skill acquisition and development of expertise [23,53,54,55,56,57]. They are sensitive to performance, subjective measures of workload, including surveys such as the NASA-TLX, and neuroimaging modalities, including fNIRS, EEG, and fMRI. Moreover, neuroadaptive training incorporating both behavioral and cognitive workload measures improves the efficiency of skill acquisition over multiple sessions [57]. Looking into the effects of flight-simulator training on deep brain areas, previous studies utilizing an aerial pursuit task inside an fMRI have found that the cerebellum, basal ganglia, and cortex have significantly differential activation relating to task performance [58]. Their activation changes in response to either increasing or decreasing performance based on the match of skill level to task difficulty, making them prime targets to seek out the effects of neuromodulation on training.

In this study, our goal is to determine the effects of targeted electrical neurostimulation to the right DLPFC, an area associated with skill in successfully flying an aircraft [58], as well as perceptual–motor dual tasks [32,33], and investigate the effects on both behavioral performance and brain function. The DLPFC is known to play a role in many cognitive functions such as working memory [59], attention [60,61], and motor planning [60] which are all relevant to tasks encompassing the ability to pilot an airplane. This study is the first to our knowledge that applies tDCS and fMRI simultaneously to comprehensively assess the performance enhancement of both novice and advanced learners during flight training with a realistic and ecologically valid landing task. Findings from this study can inform applications of neurostimulation to enhance learning in a wider array of real-world tasks and other professions.

## 2. Materials and Methods

### 2.1. Participants

Twenty-six college-aged students (ages 18–22, eighteen male) with experience piloting gliders were recruited for this study (experience ranges from 1–130 h, mean 14 h). Participants confirmed to be proficient in English and had a vision at or corrected to 20/20. All participants signed written informed consent in accordance with the Declaration of Helsinki. The protocol was approved by the National Institute of Information and Communications Technology (NICT) Human Subject Review Committee. Participants were given monetary compensation for their time. Two participants were excluded from the analysis due to poor performance.

Participants were randomly assigned to the active tDCS stim or the sham groups according to an order generated prior to data collection, resulting in 13 participants each (of these, one from each group was excluded as described above) resulting in 12 participants each in stimulation and sham. After data collection, participants were further grouped into novice or advanced levels based on their past number of hours flown in a glider plane. All participants were sorted into two groups around the median (4.5 h) for analysis, resulting, again, in 12 participants in novice and advanced groups each.

### 2.2. Experimental Procedure

We designed a three-run flight-simulator piloting protocol based on the findings and foundations of our previous neuroimaging and neurostimulation studies [26,27,56,62,63,64], determined an ideal target-brain region based on other tDCS piloting literature [29], and decided upon whole-brain regions of interest related to complex perceptual-motor tasks required in aviation to analyze based on recent fMRI findings [58].

**Figure 1:**
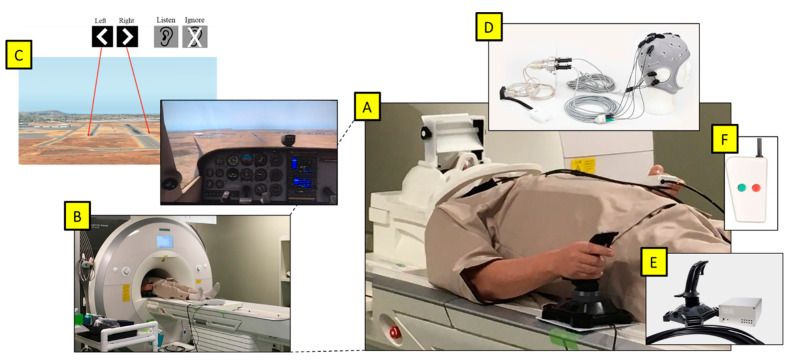
(**A**) Participant prepared for the experiment. (**B**) Magnetom Prisma 3T fMRI. (**C**) Projected viewpoint from the cockpit in the flight simulator via a mirror above eyes, with images displaying the target runway and the auditory task at the start of each trial. (**D**) Starstim fMRI-safe HD-tDCS placed on the head. (**E**) NATA Technologies fMRI Joystick in the right hand. (**F**) fMRI-safe 2-button controller for the auditory task in the left hand.

The experiment was divided into a pre-fMRI instructional section followed by three fMRI runs conducted sequentially in a single session. During the instructional section, participants signed consent forms and read instructions provided in both English and Japanese on the task. In addition, they were allowed to practice the flight-simulator task at a desk in order to acclimate to the controls for up to thirty minutes. This practice included performance feedback on the landing task to assist the participants in understanding how to improve. Following this, participants changed into MRI-safe clothes, were outfitted with the tDCS cap, and were led into the MRI room by a technician (Figure 1). Each of the three runs had two segments: a five-minute resting state scan followed by approximately a twenty-minute task scan. At the conclusion of the experimental period, a final T1 MRI scan was run for anatomical registration (Table 1). 

In this single-blinded protocol based on previous experiments [56,62], participants were randomly assigned to either the control group which received a tDCS sham, or the experimental group which received active tDCS stimulation. At the completion of the experiment, a verbal survey was conducted to ask about any effects from the tDCS that were felt such as tingling, burning, or pain, and whether they were able to tell whether they received active stim or sham. 

The three runs of this protocol were labeled as pretraining, training with feedback and tDCS, and post-training (Table 1). During the first run (pretraining), participants performed the task with no performance feedback and no tDCS. During the second run (training), participants were provided with visual performance feedback of their landings, exactly as provided during practice, as well as either sham or active stim. During the third run (post-training), tDCS was ended and there was no performance feedback. Participants were informed of when the tDCS began and ended but not which condition they were receiving.

### 2.3. Flight Landing Task

Participants operated a single-engine propeller aircraft (Cessna 172 Skyhawk) on the final approach to the runway using the flight-simulator X-Plane 11 (Laminar Research, Columbia, SC) and coded in Java to automate the protocol (Figure 1C). The initial flight speed was fixed and the throttle was set to idle. The subjects controlled the pitch, roll, and yaw of the plane with a single joystick in the right hand (Figure 1E). The altitude and distance from the runway were set to allow for landing within a window of 30–45 s. The difficulty, and therefore induced cognitive workload, was modulated in three independent ways (Table 2).

First, there were wind-present and wind-absent conditions, with wind providing more difficulty. Within the wind-present conditions, there were four unique wind patterns in different directions and strengths but comparable in difficulty. The presence or absence of wind was not indicated to the participant in each trial but they were made aware that this difference existed prior to starting.

Second, participants were indicated at the start of each trial to either perform or ignore the secondary auditory task by a visual marker on the screen (described below). In every trial, the auditory stimuli would play regardless of the condition. 

Third, two parallel runways were present at the simulated airport: one narrow, and one wider with a longer tarmac leading up to the indicated landing point. At the start of each trial, an arrow visually indicated which was the target runway. Participants were instructed to land as close to the start of the runway as possible while still landing properly according to feedback provided during practice.

Each combination was provided in pseudorandom order three times per run. Feedback was provided during the practice time and the training run visually on screen for several seconds after landing while the simulator was paused. The measures used to determine performance and success of landing were the landing g-force, the force experienced by the landing gear converted into units of gravity, the landing rate, as calculated by the average feet per minute in descent speed of the last half of the landing period, and the flair, a measure of pulling up on the joystick to raise the pitch just before landing. These three measures were automatically calculated and presented on screen with text as following: (i) flair as appropriate combinations of “very good”, “good”, “poor”, “aggressive”, “early”, and “late” and (ii) the overall landing skill based on g-force and landing rate categorized into “excellent”, “good”, “acceptable”, “poor”, and “bad”. 

### 2.4. Auditory Task

A secondary task was performed as part of the dual-task paradigm to measure free workload overhead as well as induce higher workload as part of the difficulty modulation, as we described previously [65]. The task was conducted with a basic device with two buttons held in the left hand (Figure 1F). Once every two seconds, an auditory stimulus representing a callsign containing a basic color and number combination (i.e., Blue 4, Green 9) was played via earbuds provided to the participants. Participants were instructed to memorize the parity (even or odd) of the number following every target “Red” stimulus and respond with a button press to each following red callsign number based on whether the parity was a match or mismatch (i.e., Red 5 followed by Red 7 is a match, but Red 5 followed by Red 2 is a mismatch). In the ignore condition, the audio played but participants were instructed not to respond. 

### 2.5. tDCS Neurostimulation

**Figure 2:**
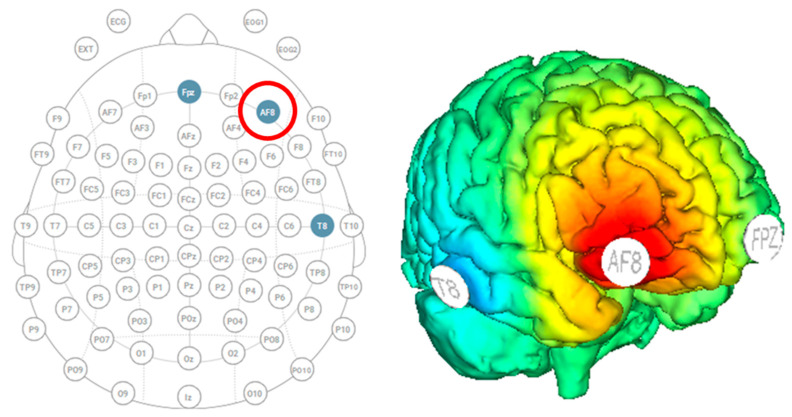
Starstim HD-tDCS with the anode over AF8 (indicated by the red circle) and cathodes over Fpz and T8 to stimulate the right dorsolateral prefrontal cortex.

Participants were outfitted with a Starstim MRI-safe high-definition tDCS (Neuroelectrics, Barcelona, Spain) with three electrodes to localize stimulation over the right dorsolateral prefrontal cortex (Figure 2). The three electrodes were 8 cm^2^ foam circles, and conductance was enhanced by saline solution. The anode stimulation was placed over AF8, and the two cathode returns were placed over Fpz and T8. The active stimulation group received thirty minutes of stimulation at 1.5 mA, including 30 s of ramp up and 30 s of ramp down. The sham group received only 30 s of ramp-up stimulation at the start and 30 s of ramp-down at the end. The head cap remained on participants throughout the entire experiment to allow them to stay within the MRI.

### 2.6. fMRI Neuroimaging

#### 2.6.1. Scanning

A Magnetom Prisma 3T scanner (Siemens Medical Solutions USA, Malvern, PA, USA) at the Center for Information and Neural Networks was used to conduct the fMRI data collection. Similar procedures were used as those reported in Gougelet et al. [58] and Durantin et al. [66]. A multiband (factor = 2) gradient-echo Echo-planar imaging (EPI) sequence was used to acquire the scans. The scanning parameters were the following: 32 channel head coil; FOV = 192 × 192mm; Matrix 64 × 64; TR = 1700 ms; TE = 30 ms; FA = 70 degrees; slice thickness = 3.0 mm no gap (3 × 3 × 3 mm voxel resolution across the entire brain); number of slices = 50; series = interleaved). Dummy scans were automatically collected by the Siemens Prisma 3T Scanner. 

The fMRI scanning consisted of three sequential runs without leaving the scanner (Table 1). Each run consisted of a resting state of approximately 5 min followed by an experimental task of approximately from 17 to 22 min (time of each session was determined by the duration it takes to land the plane, so varied across participants and runs). After fMRI scanning, a T1 anatomical MRI scan with 1 × 1 × 1 mm voxel resolution was acquired. The resting state scans were not used in this fMRI analysis. 

To reduce head and body movement artifacts during fMRI scanning, participants were instructed to keep their bodies as still as possible. In addition, cushions around the head were also used to immobilize it. The fMRI-compatible control stick (NATA technologies) was placed on the right side of the participant and the base was affixed to the scanner bed to reduce unintentional movement (Figure 1E). An fMRI-compatible button box (Current Designs—2 button) was placed in the left hand of the participant for a response during the auditory task. The video was presented to the participant by a projector to a mirror behind the head coil. The audio was presented by MR-compatible air tube insert earphones providing approximately 20 dB of passive attenuation. The average sound level of the speech presented in the left and right ears was approximately 91.3 dBA. The average sound level of the background airplane sound of the game was 76.9 dBA. The maximum sound-pressure level recorded inside the bore of the multiband EPI sequence used in this study was 95 dBA (A microphone on Opto Acoustics MRI-compatible noise-canceling headphones was used for recording).

#### 2.6.2. Preprocessing

SPM12 (Wellcome Department of Cognitive Neurology, UCL, London, UK) was used to preprocess the fMRI scans. Images from the experimental session were realigned, unwarped, and spatially normalized to a standard space using a template EPI image (2 × 2 × 2 mm voxels) provided in SPM and were smoothed using an 8 × 8 × 8 mm Full Width at Half Maximum (FWHM) Gaussian Kernel. The template image for spatial normalization was the SPM MNI EPI image (given with the SPM software). The source image for spatial normalization was the EPI image after realignment and unwarping preprocessing steps. Using the mean EPI image rather than an anatomical T1 or T2 image for normalization avoids potential errors that could arise from the additional step of coregistration that would be necessary. 

#### 2.6.3. Analysis

For each participant, a fixed-effect analysis using a general linear model GLM (SPM12) was used to determine regional brain activity for the various contrasts of interest. A mixed-block and event-related design was employed. The canonical hemodynamic response function HRF was convolved with the onset of the various events to account for lag in the blood oxygenation level-dependent (BOLD) response. Additionally, autoregression was used to correct for serial correlations and high pass filtering (cutoff period 128 s) was carried out to reduce the effects of extraneous variables (scanner drift, low frequency noise, etc.). The aircraft landing trials were modeled as blocks from the start of flying to the time of touchdown. The duration of the landing blocks varied according to piloting from approximately 30 to 45 s. There were 4 different types of landing conditions modeled depending on the presence or absence of the auditory task and the presence or absence of a crosswind making the condition relatively easy or hard (Fly_Listen_Hard, Fly_Listen_Easy, Fly_NoListen_Hard, and Fly_NoListen_Easy). Additionally, the time after landing for 4 s was modeled for these same above conditions as a feedback period for all 3 sessions even though explicit feedback is only given in run 2, the training session. The 8-s instruction period was modeled as a variable of noninterest. The audio stimuli presented for the audio task concurrently during the landing task were modeled as events in accordance with the 4 different types of landing conditions given above. Both the color and number of auditory stimuli were modeled as well as whether the stimuli were a target (red) or distractor (other color). The button press responses (same or different) were also modeled as events. In total, for each session, there were 27 conditions in addition to the 6 realignment parameters (to account for biases in head movement correlations) that were also included in the GLM. Autoregression was implemented to correct for serial correlations and high pass filtering (128 s cutoff period) was implemented to reduce the effects of extraneous variables (low frequency noise, scanner drift, etc.).

Random effects between groups’ *t*-tests were conducted for the contrasts of interest to determine significant differences between the tDCS active stim and tDCS sham groups. The primary contrasts conducted for this experiment include the following:

- Post((Fly_Listen_Hard—Fly_Listen_Easy)—(Fly_NoListen_Hard—Fly_NoLiten_Easy))—Pre((Fly_Listen_Hard—Fly_Listen_Easy)—(Fly_NoListen_Hard—Fly_NoListen_Easy)). This contrast is essentially looking at differences in brain activity post relative to pretraining for the most difficult flying condition requiring dual-task attention controlling for task and stimulus variables. It is predicted that this contrast will be most sensitive to learning effects induced by tDCS stimulation. In particular, brain regions involved with perceptual-motor control related to piloting during landing are expected to show greater differential activity between the tDCS stim and sham groups. These brain regions include the cerebellum, basal ganglia, and premotor cortex. Region-of-interest (ROI) analyses were conducted using masks constructed from the WFU PickAtlas Tool in SPM12 for the left and right cerebellum, as well as the left and right caudate of the basal ganglia. The mask used for the premotor cortex included both ventral and rostral maps given in Neubert et al. [67];
- Training((Fly_Listen_Hard—Fly_Listen_Easy)—(Fly_NoListen_Hard—Fly_NoListen_Easy)). This contrast looks at brain activity during the training session for the most difficult flying condition requiring dual-task attention controlling for task and stimulus variables. It is predicted that differences in brain regions activated to a greater extent during tDCS stimulation will be identified. In particular, the DLPFC, the site of tDCS stimulation, is predicted to show significant differential activity. The ROI map used for the DLPFC was from Sallet et al. [68] including both area 9/46 dorsal and area 9/46 ventral;
- Psychophysiological Interaction Analysis (PPI) for the Training((Fly_Listen_Hard—Fly_Listen_Easy)—(Fly_NoListen_Hard—Fly_NoListen_Easy)) Contrast. Standard procedures in SPM12 were used for the PPI analysis. This included first extracting the neural activity of the seed voxel (VOI) in the DLPFC (MNI 34,46,28) by deconvolution with the hemodynamic response for the contrast listed above using the PPI function in SPM. The results of this analysis are three files: PPI.ppi (the interaction term created by the PPI analysis), PPI.Y (The time-series extracted from the VOI), and PPI.P (the convolved onset times). These three files together with the 6 realignment parameters (variables of noninterest) are then used as regressors in an SPM fixed-effect analysis separately for each participant. The resultant PPI contrast image for each participant is used in the between groups’ (tDCS stim vs. sham) random-effects analysis. ROI analyses were conducted assessing the connectivity from the site of stimulation, DLPFC, to brain regions predicted to be involved with perceptual-motor control of airplane piloting during landing (cerebellum, basal ganglia, and premotor cortex);
- Training(Aud_Listen_Hard—Aud_Listen_Easy). It is hypothesized that tDCS will act to focus brain processes involved with the primary training task (in this case the airplane landing task) and suppress brain processes involved with other tasks (e.g., the auditory response task). It is therefore predicted that when the flying task is more difficult there will be greater suppression of auditory (BA41 and 42) and speech (premotor cortex, Broca’s area) processing regions in the brain for the active stimulation over the sham group. The ROI for the auditory cortex was made from the WFU PickAtlas Tool in SPM12 including Brodmann Area 41 and 42 with a dilation of 1. The ROI map for the dorsal premotor cortex PMd was from Sallet et.al. [68]. The ROI map for Broca’s area was defined as BA44 ventral by Neubert et al. [67].

### 2.7. Behavioral Performance Measures and Analysis

The following measures were analyzed for the primary landing task: landing g-force and landing rate. Each performance measure was baselined within subjects by subtracting the average of the pretraining run performance for each trial type (Table 2). 

For the secondary auditory task (which includes half of the total trials), the following measures were analyzed: accuracy (percentage correct of all target stimuli), incorrect percentage (percentage incorrect response of all target stimuli), false-positive rate (percentage of responses to nontarget stimuli), and false-negative rate (percentage of missed responses to target stimuli).

The primary analysis was performed using T-tests across all subjects for the training and post-training runs versus the pretraining. Further detailed statistical analysis was conducted using linear mixed models (LMM) in NCSS 2019. The landing-task performance measures were analyzed using *run* (training vs. post), *tDCS* condition (active vs. sham), and *experience* (novice vs. advanced) as main factors and *runway* (wide vs. narrow), *wind* (present vs. absent), *auditory task* (perform vs. ignore), and *pretraining performance* (continuous data) as covariates. The subject was included as a random factor and the best covariance pattern was determined by minimizing the Akaike information criterion (AIC) value. The full model was run + tDCS + experience + run*tDCS + experience*tDCS + runway + wind + auditory + run 1 performance. Post hoc comparisons were performed for all pairs of factor levels and multiple comparisons were corrected with the Bonferroni method.

In addition, training and postruns were independently analyzed using the model tDCS + experience + experience*tDCS + runway + wind + auditory + pretraining performance. 

The secondary auditory-task performance measures were likewise analyzed using the following LMM models: for training and postruns together, run + tDCS + experience + run*tDCS + experience*tDCS + runway + wind + pretraining performance. For training and post independently, tDCS + experience + experience*tDCS + runway + wind + pretraining performance.

## 3. Results

### 3.1. Behavioral Performance Measures 

**Figure 3:**
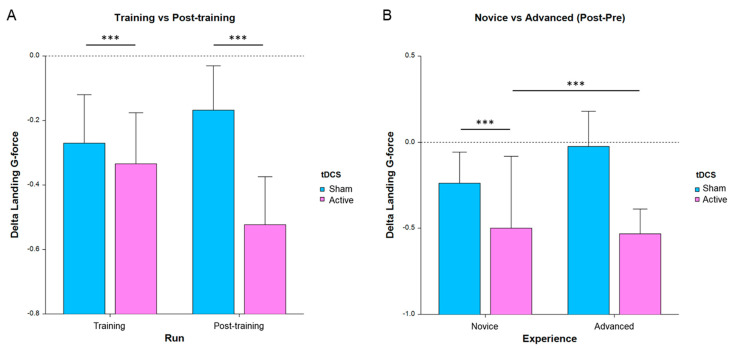
Average change in landing g-force for all subjects, where lower bars indicate a greater improvement in performance from run 1 (*** *p* < 0.001, error bars are SE). (**A**) Performance separated by run and tDCS condition. (**B**) Post-pretraining separated by experience and tDCS condition.

For this analysis, landing g-force and landing rate were the primary measures of interest, as those were used to provide feedback during practice and tDCS-feedback segments. In particular, landing g-force was found to provide the most sensitivity for the main effects and interactions of the LMM models. One-sided post hoc T-tests on the change in performance compared to pretraining (Figure 3A) revealed a significant improvement (Bonferroni corrected) for the stimulation group during training (T_288_ = −2.12, *p* = 0.035 corrected) and post-training (T_287_ = −3.51, *p* = 0.0005 corrected) but not for the sham group. Figure 3 below depicts the improvement in training and post-training and for each group. The complete results are in Table 3.

Fewer main-effect significances were found for the landing rate measure and are included in the appendix (Table S1). In particular, we found a significant main effect of experience for post-pre (*p* < 0.05) and post hoc comparisons showed a significant difference between novice and advanced for active stim (*p* < 0.05). In the training session, there was a main effect of experience (*p* < 0.01) and interaction between experience and tDCS condition (*p* < 0.05). Post hoc comparisons showed a difference between novice and advanced for active stim (*p* < 0.05) and within novices between active stim and sham (*p* < 0.01). 

Secondary auditory-task performance was analyzed with LMM as described in the methods section; however, no significant results were found for any main effects or interactions in any of the measures, including accuracy, incorrect percentage, false positives, and false negatives.

In summary, we found important performance differences in multiple relevant indices of safe and successful landing task measures that were affected by tDCS stimulation and differentially applied to those with higher or lower experience levels. We also found that the wind condition had the strongest effect on these behavioral measures.

### 3.2. Brain Function Measures

**Figure 4:**
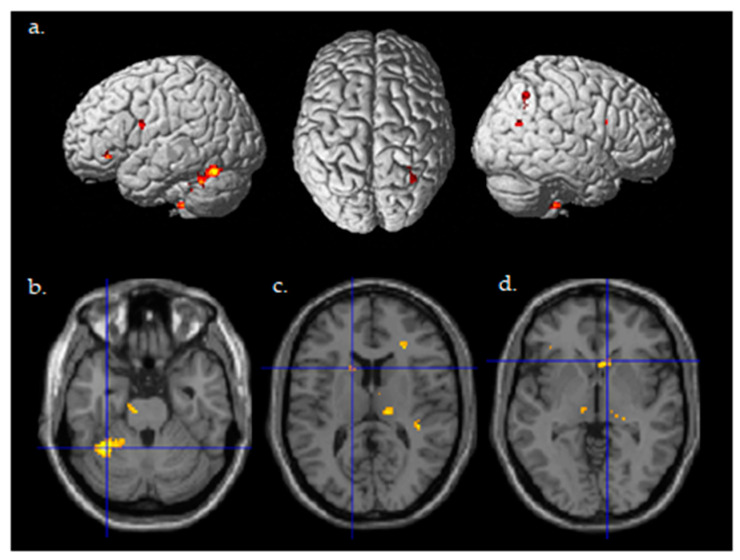
Differential brain activity for the active Stim > Sham group for the contrast focusing on selective attention task-dependent learning-related activity Post((FlyLsnHard-FlyLsnEasy)—(FlyNoLsnHard-FlyNoLsnEasy))—Pre ((FlyLsnHard-FlyLsnEasy)—(FlyNoLsnHard-FlyNoLsnEasy)) *p* < 0.001 uncorrected. (**a**) Rendered on the surface of the brain’s left, top, and right views. (**b**) Horizontal slice through the cerebellum (centered at MNI −32,−50,−26), ROI analysis for left cerebellum pFWE < 0.05 corrected for multiple comparisons. (**c**) Horizontal slice through caudate of basal ganglia (centered at MNI -14,14,12), ROI analysis for left caudate pFWE < 0.05 corrected for multiple comparisons. (**d**) Horizontal slice through caudate of basal ganglia (centered at MNI 10,20,−2), ROI analysis for right caudate pFWE < 0.05 corrected for multiple comparisons.

A random effects two-sample *t*-test analysis for the tDCS active stim group > tDCS sham group was conducted using the contrast images of the SPM analysis for each participant for the contrast of Post((Fly_Listen_Hard—Fly_Listen_Easy)—(Fly_NoListen_Hard—Fly_NoListen_Easy))—Pre((Fly_Listen_Hard—Fly_Listen_Easy)—(Fly_NoListen_Hard—Fly_NoListen_Easy)). This contrast is essentially investigating changes in brain activity post relative to pretraining that are present during the most difficult flying condition during the dual-task condition requiring attention to both flying and the audio task. The group that received active stimulation during the training session showed significant differential activity in various brain regions shown in Table 4 and Figure 4 (threshold set at *p* < 0.001 uncorrected with voxel extent threshold of 15). It should be noted that the extent threshold was used primarily for visualization purposes and was not used for subsequent ROI statistical analyses correcting for multiple comparisons. Of primary interest is the differential activity found in the left cerebellum, the left caudate, and the right caudate that was found to be significant (pFWE < 0.05 corrected for multiple comparisons, Figure 4b–d, Table 5) using ROI analysis (see methods for ROI maps used). No significant differences were present for the tDCS sham > tDCS stim analysis using a threshold of *p* < 0.001.

**Figure 5:**
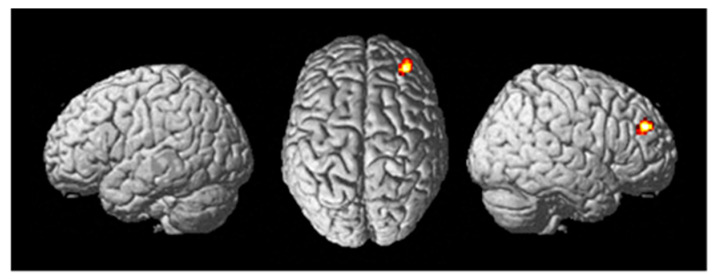
Differential brain activity for the tDCS stim > sham group for the contrast focusing on selective attention task-dependent activity during training tDCS session training ((FlyLsnHard-FlyLsnEasy)—(FlyNoLsnHard-FlyNoLsnEasy)) *p* < 0.001 uncorrected. Rendered on the surface of the brain’s left, top, and right views. The focus of differential activity is centered at MNI 34,46,28, ROI analysis for right DLPFC (using anatomical mask from Sallet et al., 2013) pFWE < 0.05 corrected for multiple comparisons.

A random effects two-sample *t*-test analysis (active stim group > sham group) during the training session (in which tDCS was administered) across the contrast images of each participant for the following contrast ((Fly_Listen_Hard—Fly_Listen_Easy)—(Fly_NoListen_Hard—Fly_NoListen_Easy)) was conducted. This contrast is meant to focus on the most difficult flying condition that also requires dual-task attention. The primary brain region showing differential activity for this contrast between active stim and sham groups was the DLPFC (See Table 6 and Figure 5) (threshold set at *p* < 0.001 uncorrected with voxel extent threshold of 15). The intended site of tDCS stimulation was also the DLPFC. ROI analysis (see methods for ROI map used) showed significant differential activity in the DLPFC when correcting for multiple comparisons (See Table 7, pFWE < 0.05). No significant differences were present for the tDCS sham > tDCS stim analysis using a threshold of *p* < 0.001.

**Figure 6:**
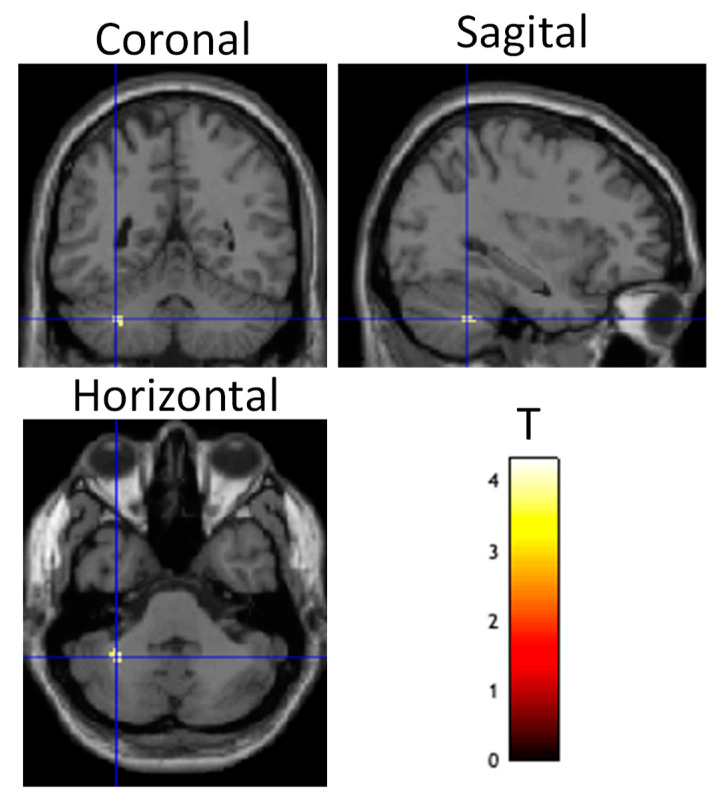
Differential brain activity for tDCS stim > sham group for the psychophysiological interaction connectivity analysis using the DLPFC as the seed ROI and the contrast focusing on selective attention task-dependent activity during training tDCS session training ((FlyLsnHard-FlyLsnEasy)—(FlyNoLsnHard-FlyNoLsnEasy)) *p* < 0.001 uncorrected. Differential activity is shown on coronal, sagittal, and horizontal slices through the cerebellum (centered at MNI −32, −50, −40). Spherical small volume correction analysis centered at MNI 32, −50, −26 with a radius of 15mm showed significant differential connectivity between DLPFC and cerebellum pFWE < 0.05 corrected for multiple comparisons.

A PPI analysis was conducted to determine the contrast-dependent connectivity during the training session from the DLPFC to the rest of the brain. The contrast used in the PPI analysis was the same as utilized above ((Fly_Listen_Hard—Fly_Listen_Easy)—(Fly_NoListen_Hard—Fly_NoListen_Easy)). The PPI contrast image for the participants was used for a random-effects two-sample *t*-test analysis (active stim group > sham group). Differential connectivity during the training session between the two groups was present from the DLPFC to a few brain regions, including the cerebellum (See Table 8) (threshold set at *p* < 0.001 uncorrected with voxel extent threshold of 15). Although the ROI analysis over the entire left cerebellar hemisphere did not reach significance pFWE < 0.05 when correcting for multiple comparisons, a small volume correction for multiple comparisons analysis using the peak voxel in the cerebellum from the learning-related contrast given in Table 5 with a radius of 15mm did show significant differential connectivity (pFWE < 0.05) for active stim > sham (Figure 6, Table 9). No significant differences were present for the sham > active stim analysis using a threshold of *p* < 0.001.

**Figure 7:**
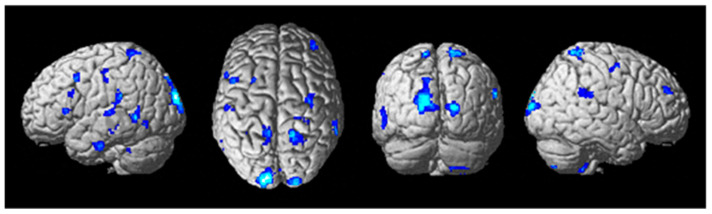
Differential brain activity for the tDCS stim < sham group for the contrast focusing on the auditory task during difficult compared to easier flying conditions during the training tDCS session Training(SndLsnHard-SndLsnEasy) *p* < 0.001 uncorrected. Rendered on the surface of the brain’s left, top, and right views. ROI analyses in the left primary auditory cortex, left dorsal premotor cortex, and ventral Brodmann area 44 showed significant differential activity pFWE < 0.05 corrected for multiple comparisons.

To determine potential suppression of auditory and speech-processing areas as a result of tDCS stimulation, a two-sample *t*-test of tDCS active stim < sham during the training session (in which tDCS was applied) was conducted for contrast images of the participants (Aud_Listen_Hard—Aud_Listen_Easy). The results of the analysis showed a large number of brain regions that have significantly less activity for the active stim group relative to the sham group (See Figure 7, Table 10) (threshold set at *p* < 0.001 uncorrected with voxel extent threshold of 15). Of particular interest is the finding of significantly less differential activity (pFWE < 0.05 corrected for multiple comparisons) for the ROI analyses in the left auditory cortex, the dorsal premotor cortex, and Broca’s area (Table 11). No significant differences were present for the active stim > sham analysis using a threshold of *p* < 0.001.

## 4. Discussion

In this study, our aim was to assess the impact of tDCS stimulation on the brain and behavioral performance in a flight simulator for pilots conducting a realistic landing task. Participants were first randomly assigned to one of two groups: the control group receiving sham tDCS and the experimental group receiving tDCS stimulation to the right dorsolateral prefrontal cortex, an area associated with the level of performance skill during flight. For our analysis, we split all of the pilots into two equally sized groups around the median of previous hours of flight experience, making the novice and advanced groups. Our experimental design allowed us to elucidate the effects of neurostimulation with performance feedback during training, as this protocol of only providing specific numerical and contextual feedback in the second of three runs has previously been demonstrated to achieve good results. We targeted the right DLPFC as it is specifically recruited during difficult flight tasks to analyze changes in functional regions of interest as well as connectivity between the area of stimulation and other regions of interest (ROI). In addition, we determined the effect this had on brain areas related to the secondary auditory task performed in half of all trials, a condition designed to replicate receiving auditory instructions and warnings while visually and tactilely controlling a plane. We found that tDCS stimulation improved the task performance (landing of aircraft on a runway) during and post-training, particularly in novice pilots, a finding consistent with established literature regarding the learning of new skills. Moreover, the stimulation group demonstrated increased neural activity in the left cerebellum and left and right basal ganglia caudates post-training relative to pretraining for the most difficult task conditions. The stimulation group also showed increased brain activity during training at the stimulated area of the right DLPFC, as well as higher functional connectivity to the left cerebellum. Finally, the stimulation group had inhibited activity to the left primary auditory cortex, left dorsal premotor area, and Broca’s area, specifically during the secondary auditory task. These points are discussed in further detail below.

### 4.1. Effects on Behavioral Performance and Expertise

Performance feedback provided during the familiarization period pre-fMRI, as well as during the training session (run 2) at the end of each landing trial, was written in simple terms (from excellent to bad, color-coded with blue, green, yellow, orange, and red) and was calculated automatically based on the landing g-force, landing rate, and flair to match realistic values. This feedback design, including the pretraining, training, and post-training protocol, has been used in previous tDCS-fMRI experiments and has been found to elicit performance increases and performance-related enhancements in brain connectivity [26]. In order to focus on the performance improvement, rather than preexisting intersubject skill levels, data from runs 2 and 3 were baselined for each participant and each difficulty condition to the respective performance from run 1 (Table 2). This allowed us to correct for each unique trial and focus on performance changes.

The primary performance measure that displayed significant main effects was the landing g-force, which is indicative of the deceleration that the aircraft experienced at the point of landing (Figure 3, Table 3). As stated, participants were instructed to land as softly as possible and received feedback on this specifically, which directed their skill increases. The tDCS active stimulation vs. sham showed a significant group difference and post hoc tests revealed that novice pilots received a greater benefit than advanced pilots, which is consistent with what is known about learning new skills. We found that performance improvement also significantly changed based on experience level and the interaction between experience and tDCS condition. These results are in line with previous findings regarding the increased benefits of tDCS training on lower-skill or less-experienced performers [17,45,46]. In practical application, this indicates that neurostimulation is most effectively applied early on in training, rather than with more experienced pilots. We were also able to determine that only the wind condition, not the size of the runway or the performance of a secondary auditory task, significantly affects landing performance. This may inform what conditions pilots should be more aware of during flight compared to others and indicate what type of training is more important. There are many other environmental conditions as well that may be tested in the future, including time of day (light and darkness), fog, temperature, altitude of the airport, weather conditions including rain and snow, or even different weights of the cargo (whether passengers or freight). The experimental protocol developed here can test for these covariates and inform the direction of training, and what conditions to focus neurostimulation sessions on.

The landing rate measure showed similar, but less significant, differences for the same conditions (Table S1). This indicates that the moment of landing, rather than the path flown through airspace, is more affected by the mental workload induced by more difficult conditions. We also examined a large number of additional performance measures, including total trial time, time to land, time to first touchdown, time between touchdown and landing, speed at landing, pitch at landing, total air distance, total pitch, pitch deviation through flight, roll deviation through flight, and yaw deviation through flight, distance from the start of the runway, and horizontal distance from the runway centerline. However, none of these were found to have significant main effects, and their mention here is intended to guide future studies in flight simulators. Taken in the context of the previous significant results, we can posit that only when direct feedback is provided to operators about points of improvement or goalposts to reach to meet certain task demands are they able to improve those specific metrics. In order to improve these other metrics which may be just as important, such as landing aligned with the runway near the start of the tarmac, feedback to these must be specifically provided during training. It is important to keep in mind, however, that overloading the pilot may have the opposite effect. To keep operator workload at an optimal level, only the most important requirements should be trained at one time until experience is gained.

We did not find any significant main effects or interactions in the secondary task (auditory) results. This may be due to the task itself not providing enough challenge, therefore causing a ceiling effect of performance regardless of experience or landing task conditions. This includes the lack of tDCS-related changes in performance, which does follow our expectations as we were not stimulating an area of the cortex involved in auditory processing. From a positive point of view, we saw no signs of inattentional deafness, which occurs when attentional demands and stress are so high that the brain’s perception of peripheral information shuts down [66]. However, this is not entirely directly applicable to our protocol, as we specifically instructed participants to attend to the auditory task second after making certain the primary flight task was performed correctly, whereas negative repercussions of inattentional deafness occur when the peripheral information, i.e., auditory alarms of danger, require primary attention.

### 4.2. Brain Activity and Connectivity Modulation

From the fMRI data, we examined the change in brain activity across task conditions before and after training. ROI analysis of active stim minus sham between post- and pretraining runs (Figure 4), shows significantly more activation in the left and right basal ganglia caudates as well as the left cerebellum. Previous research using flight simulators suggests that the basal ganglia is correlated with high flight performance [66]. This matches the behavioral results in which the tDCS stimulation group significantly increases their performance more than the sham group from pre- to post-training runs. Brain activity results confirm the task performance results while demonstrating that alternate perceptual-motor skills such as our landing task follow established literature. The basal ganglia is thought to be involved with reinforcement-based learning [69] and the cerebellum is thought to be involved with error-feedback-based learning [69] and instantiation of internal models for perceptual-motor control [70,71,72,73,74]. The findings of greater cerebellum and basal ganglia activity post-relative to pretraining for the tDCS stim group are consistent with enhanced learning and acquisition of perceptual-motor internal models involved with landing an airplane. It should be noted that the combination of the basal ganglia, cerebellum, and cortex network is active across a range of important motor tasks involving action selection and specification, and the decisions of what to do and how to do it [75]. This naturally comes into play during high-pressure, fast-reaction situations such as landing a plane.

The cerebellum is involved in error-based feedback training [69,74], becoming more active when incorporating past mistakes into planning for future action [62]. This aligns with our training protocol in which landing performance was graded and provided to each participant after every trial during the second run. By priming pilots with an easy-to-understand evaluation of landing success, they were better able to self-evaluate even when it was removed again post-training. This same region was found to be involved during the training period as well and the importance is described below.

During training, the right DLPFC displayed higher task-related activity in the stimulation group (Figure 5). This was the area of tDCS stimulation, which follows that it would be more active during this period, but that is not the entire story. Notably, the psychophysiological interaction connectivity analysis (PPI) found significant functional connectivity between the right DLPFC and the left cerebellum (Figure 6), the same area that was more active in the stimulation group post-training. This implies that the effects of tDCS resulted in the establishment of perceptual-motor internal models important for increased landing task performance (Figure 3). However, there was no significant functional connectivity between the DLPFC and the caudate, despite the basal ganglia displaying higher activity post-training in the same pilots. The caudate is, therefore, not directly influenced by tDCS as evaluated by PPI analysis during the training session but may, nevertheless, reflect the resulting performance improvement of the tDCS stim group post-training. This makes sense as it is part of the cerebellum–basal ganglia–cortex network described above. In addition to being directly involved in higher behavioral performance, the basal ganglia is also associated with the inhibition of nontask-related neural processes [58]. By minimizing extraneous mental load from unrelated sensory input or other distractors, more working memory can be devoted to the flight task. In our experiment, this potentially directed focus away from the secondary auditory task, including “ignore” trials where the sound could be a distractor, further improving performance.

Although we did not find any differences in behavioral performance of the secondary auditory task that was attended to in half of all trials, we did find a significant decrease in activity for the active stim group during the training run in several areas including the left primary auditory cortex, the left dorsal premotor cortex, and the ventral Brodmann area 44 (Broca’s area) (Figure 7). These changes, as we predicted, indicate inhibition in auditory and speech-related functional regions [13], which were specifically attended during the performance of the secondary task. This shifting of mental resources away from the auditory task to the landing task may aid in improving performance in the primary goal of landing safely and successfully. It was very interesting how these changes were the result of anodal stimulation to the right DLPFC and not cathodal inhibitory stimulation elsewhere. This demonstrates that there may be task-specific and unintended effects of tDCS application during training. However, this may inadvertently also lead to inattentional deafness, a major cause of accidents in high-stress situations [66]. Keeping a balance of improved performance and speed of skill acquisition while not inhibiting other necessary processes will be vital to the real-world application of tDCS in pilot training. In the case of this protocol, providing feedback to the auditory task and stating that it was of equal importance to the landing may have mitigated these inhibitory effects. We can see that not just stimulation, but also context and the intention of the learner, are what guide the neural effects of training on the brain.

### 4.3. Limitations

We recruited a sample size of twenty-six experienced glider pilots for this study and were able to use twenty-four of them for the analysis. This relatively small size was due to a number of factors, including the availability of pilots in our area included under the Human Subject Review Committee’s supervision (accounting for a small sample pool and long-distance travel to the laboratory), the difficulty of securing dedicated time to use the fMRI at our research institute, and finally, the onset of COVID-19 towards the end of data collection, prevented further safe human-subjects testing. We acknowledge these limitations and their effects on lowering the overall statistical power of our analyses and hope that our findings lead the way for larger-scale experiments.

In addition, due to the same limitations discussed above, we were restricted to a single session for each participant, broken up into three distinct runs conducted sequentially. This was also done by following a previous experimental protocol [26] using simultaneous tDCS and fMRI which identified neurostimulation-induced changes in performance and brain activity with twenty-eight total subjects, only four more than our protocol. While our findings presented here do apply to a relatively short training period, we would like to conduct a longer-term study, as the effects of tDCS have been shown to increase over repeated application [25,29]. 

We targeted the right dorsolateral prefrontal cortex in this study, which had previously been examined in another flight-simulator protocol [29]. However, other studies that targeted the left DLPFC [28], right ventrolateral PFC [27], or the motor cortex [29,46] have all had promising results in other cognitive and perceptual-motor tasks. Another promising direction to further our findings would be to compare multiple areas of stimulation with a control group to determine the most effective way to enhance learning.

## 5. Conclusions

We investigated the effects of tDCS neurostimulation to the right DLPFC (an area associated with higher executive processing and skill acquisition) on flight task performance, functional brain activity, and connectivity using fMRI. We found that the active stimulation (compared to the sham) group improved more during the training period (with performance feedback) and retained this skill increase even after tDCS with no feedback (post-training session). In particular, novice pilots received a greater performance benefit than more advanced pilots from the tDCS. The stimulation resulted in increases in brain activation in the left cerebellum and basal ganglia caudates for the most difficult conditions, areas associated with increased skill. Functional connectivity analysis showed a direct relationship between the right DLPFC and left cerebellum, elucidating the influence of tDCS. Finally, we identified auditory and speech-processing regions of the brain that were inhibited during active stimulation, effectively increasing focus on the main task (instead of the secondary task). Overall, these results illuminate a path forward for the real-world application of neurostimulation to enhance skill acquisition in the field of aviation, and also fortify a format of feedback-based training that can be applied to a wide variety of other fields requiring high perceptual-motor skills. This study further contributes to the neuroergonomics approach to the investigation of performance in aviation [54,76,77].

## Figures and Tables

**Table 1:** Experiment protocol stages and timeline, whether performance feedback was provided, and tDCS applied. All stages were in MRI: Runs 1–3 with T2 scans and T1 scan for anatomical registration at the end after Run 3.

Run 1		Run 2		Run 3	
Rest	Pre-Training	Rest	Training	Rest	Post-Training
5 min	20 min	5 min	20 min	5 min	20 min
No feedback		Performance feedback		No feedback	
No tDCS		Active Stim/Sham tDCS		No tDCS	

**Table 2:** Experiment conditions and levels in each condition. Each of the three difficulty conditions was mixed to form eight unique combinations of trials and performed three times each, for a total of 24 trials per run, 72 trials in total. For reference, Type 1 is all easy (lowest workload) and Type 2 is all hard (highest workload). Hard conditions are highlighted in bold.

	Wind Condition	Auditory Task	Runway Condition
Type 1	Absent	Ignore	Wide
Type 2	**Present**	**Perform**	**Narrow**
Type 3	Absent	**Perform**	**Narrow**
Type 4	**Present**	Ignore	Wide
Type 5	Absent	**Perform**	Wide
Type 6	**Present**	Ignore	**Narrow**
Type 7	Absent	Ignore	**Narrow**
Type 8	**Present**	**Perform**	Wide

**Table 3:** Landing g-force LMM significance results for all main effects, interactions, and covariates, as well as post hoc comparisons (* *p* < 0.05, *** *p* < 0.001).

Term	Factor	F-Ratio		*p*-Value	Partial η2
**Landing G-force Main Effects (Post-Pre)**					
	tDCS	16.78	_1/23.7_	0.0004 ***	0.415
	Experience	30	_1/24.5_	0.00001 ***	0.550
	Experience*tDCS	14.6	_1/21_	0.0001 ***	0.410
	Runway	3.02	_1/537_	0.08	0.006
	Wind	62.03	_1/548_	0.00000 ***	0.102
	Auditory	0.53	_1/537_	0.47	0.001
	Pretraining Performance	270.6	_1/489_	0.00000 ***	0.356
**Landing G-force Post Hoc Comparisons (Post-Pre)**					
	Active stim, experience novice vs. advanced	30.50	_1/25.8_	0.00002 ***	0.542
	Experience novice, active stim vs. sham	20.30	_1/25.2_	0.0003 ***	0.446
**Landing G-force Main Effects (Training-Pre)**					
	tDCS	31.9	_1/23.3_	0.00001 ***	0.578
	Experience	27.4	_1/24.1_	0.00000 ***	0.532
	Experience*tDCS	19.1	_1/20.3_	0.0003 ***	0.485
	Runway	0.67	_1/540_	0.41	0.001
	Wind	46.1	_1/552_	0.00000 ***	0.077
	Auditory	3.47	_1/540_	0.06	0.006
	Pretraining Performance	238	_1/480_	0.00000 ***	0.331
**Landing G-force Post Hoc Comparisons (Training-Pre)**					
	Active stim, experience novice vs. advanced	55.9	_1/24.3_	0.00000 ***	0.697
	Experience novice, active stim vs. sham	43.0	_1/23.7_	0.0000002 ***	0.645
**Landing G-force Main Effects (Training and Post-Pre)**					
	Run	0.06	_1/1102_	0.8	0.000
	tDCS	34	_1/22.3_	0.00007 ***	0.604
	Experience	54.7	_1/22.9_	0.00000 ***	0.705
	Run*tDCS	1.67	_1/1102_	0.2	0.002
	Experience*tDCS	23.2	_1/20.3_	0.0001 ***	0.533
	Runway	0.25	_1/1103_	0.61	0.000
	Wind	107.7	_1/1118_	0.00000 ***	0.088
	Auditory	0.9	_1/1103_	0.34	0.001
	Pretraining Performance	501.7	_1/948_	0.00000 ***	0.346
**Landing G-force Post Hoc Comparisons (Training and Post-Pre)**					
	Run 2, tDCS stim vs. sham	27.6	_1/39.6_	0.00001 ***	0.411
	Run 3, tDCS stim vs. sham	16.6	_1/39.6_	0.0004 ***	0.295
	Active stim, experience novice vs. advanced	60.4	_1/24.3_	0.00000 ***	0.713
	Experience novice, active stim vs. sham	43.0	_1/23.8_	0.000002 ***	0.644

**Table 4:** Post—Pre (FlyLsnHard-FlyLsnEasy)—(FlyNoLsnHard-FlyNolsnEasy) stim–sham.

Brain Region	Cluster Size	MNI Coordinate x, y, z	T	*p*
**Cerebellum**	447	−32, −50, −26	5.32	0.0000028
**Cerebellum**	77	8, −52, −52	4.52	0.000085
**Midbrain/Brain Stem**	69	−8, −18, −20	4.42	0.00011
**Midbrain/Brain Stem ^1^** **Hippocampus** **Thalamus**	272	12, −24, −10 24, −28, −10 16, −22, 8	5.94 4.48 4.39	0.0000027 0.000093 0.00012
**Hippocampus**	79	−36, −18, −12	5.05	0.000024
**Thalamus**	24	−8, −16, 0	4.27	0.00036
**Thalamus**	34	8, −6, 16	4.15	0.00021
**Caudate**	27	−14, 14, 12	4.21	0.00018
**Caudate**	27	6, 20, −2	4.08	0.00025
**Frontal Operculum**	20	28, 32, 12	4.05	0.00046
**Orbital IFG**	29	−42, 32, −8	4.46	0.000098
**Opercular IFG**	24	40, 8, 24	3.92	0.00092
**Medial Orbital Gyrus**	50	14, 6, −20	5.36	0.000011
**Premotor Cortex/Precentral Gyrus**	21	−44, 2, 22	4.47	0.000095
**Postcentral Gyrus**	21	42, −10, 24	3.94	0.00035
**Parietal Operculum**	69	36, −32, 26	4.99	0.000027
**Precuneus/Posterior Cingulate Gyrus**	36	18, −40, 42	4.54	0.00008
**Posterior Cingulate Gyrus**	20	−12, −40, 20	4.54	0.00008
**Superior Parietal Lobule** **Angular Gyrus**	145	32, −54, 44 38, −5, 34	4.5 3.98	0.00009 0.00031
**Middle Occipital Gyrus**	20	42, −66, 20	4.23	0.00017
**Lingual Gyrus**	21	6, −74, −8	4.12	0.00022
**Primary Auditory/Planum Temporal**	55	36, −28, 6	4.34	0.00013
**Planum Temporal**	85	−42, −34, 4	5.21	0.000016

**^1^** Mid-Brain and Brain-Stem Regions including the Substantia Nigra, Red Nucleus, Lenticular Fasciculus, and medial lemniscus. IFG = Inferior Frontal Gyrus.

**Table 5:** Post-Pre (FlyLsnHard-FlyLsnEasy)—(FlyNoLsnHard-FlyNolsnEasy) stim-sham, ROI Analysis. Note: * Significant at pFWE < 0.05 corrected for multiple comparisons within the region of interest.

Brain Region	Cluster Size	MNI Coordinate x, y, z	T	*p*	pFWE_corr
**Cerebellum**	265	−32, −50, −26	5.32	0.0000028	0.026 *
**Cerebellum**	77	8, −52, −52	4.52	0.000085	0.110
**Caudate**	27	−14, 14, 12	4.21	0.00018	0.025 *
**Caudate**	23	6, 20, −2	4.08	0.00025	0.032 *
**PMv**	2	−44, 4, 20	3.75	0.00056	0.063

**Table 6:** Train (FlyLsnHard-FlyLsnEasy)-(FlyNoLsnHard-FlyNolsnEasy) stim–sham. DLPFC = Dorsolateral Prefrontal Cortex.

Brain Region	Cluster Size	MNI Coordinate x, y, z	T	*p*
**DLPFC**	131	34, 46, 28	5.71	0.0000049
**Middle Cingulate Gyrus**	67	8, 4, 40	4.56	0.000077

**Table 7:** Train (FlyLsnHard-FlyLsnEasy)-(FlyNoLsnHard-FlyNolsnEasy) stim–sham, ROI analysis. Note: * Significant at pFWE < 0.05 corrected for multiple comparisons within the region of interest.

Brain Region	Cluster Size	MNI Coordinate x, y, z	T	*p*	pFWE_corr
**DLPFC**	94	34, 46, 28	5.71	0.0000049	0.004 *

**Table 8:** Train (FlyLsnHard-FlyLsnEasy)—(FlyNoLsnHard-FlyNolsnEasy) stim–sham, seed voxel in DLPFC (MNI 34,46,28). MTG = middle temporal gyrus.

Brain Region	Cluster Size	MNI Coordinate x, y, z	T	*p*
**Cerebellum**	18	−32, −50, −40	4.03	0.00028
**Brain Stem**	42	−8, −28, −36	4.30	0.00014
**Occipital Pole**	26	20, −94, 2	3.84	0.00044
**MTG**	26	60, −40, −12	3.86	0.00042

**Table 9:** PPI Training (FlyLsnHard-FlyLsnEasy)—(FlyNoLsnHard-FlyNolsnEasy) stim–sham, seed voxel in DLPFC (MNI 34, 46, 28), ROI Analysis. * Significant at pFWE < 0.05 corrected for multiple comparisons within the region of interest ROI. **^1^** Cerebellum ROI covering the entire left hemisphere. **^2^** Cerebellum ROI centered at MNI coordinate −32, −50, −26 with a radius of 15mm.

Brain ROI	Cluster Size	MNI Coordinate x, y, z	T	*p*	pFWE_corr
**Cerebellum ^1^**	18	−32, −50, −40	4.03	0.00028	0.211
**Cerebellum ^2^**	17	−32, −50, −40	4.03	0.00028	0.042 *

**Table 10:** Training (SndLsnHard-SndLsnEasy) sham–stim. * Cluster wise corrected for multiple comparisons pFWE < 0.05, ** Voxel wise corrected for multiple comparisons pFWE < 0.05.

Brain Region	Cluster Size	MNI Coordinate x, y, z	T	*p*
**Cerebellum**	37	30, −74, −54	4.48	0.000093
**Cerebellum**	41	−16, −56, −22	4.40	0.00011
**Cerebellum**	35	28, −42, −56	4.14	0.00022
**Cerebellum**	30	−32, −50, −36	4.09	0.00024
**DLPFC**	42	40, 50, 30	4.43	0.00011
**Anterior Insula**	22	28, 18, 10	4.45	0.0001
**IFG**	33	−50, 24, 8	4.27	0.00016
**IFG**	20	−50, 16, 26	4.24	0.00017
**Middle Frontal Gyrus**	46	−26, 10, 40	4.68	0.000057
**Premotor Cortex**	48	−48, 10, 44	4.41	0.00041
**Precentral Gyrus**	91	34, −12, 50	5.39	0.00001
**Medial Precentral Gyrus** **SMA** **Middle Cingulate Gyrus**	145	10, −20, 50 10, −2, 46 8, −12, 44	6.16 4.85 4.18	0.0000017 0.000038 0.00019
**Postcentral Gyrus**	30	−48, −20, 42	4.34	0.00013
**Postcentral Gyrus**	20	20, −34, 56	3.80	0.0005
**Postcentral Gyrus**	19	30, −34, 50	4.00	0.0003
**Medial Postcentral Gyrus** **Superior Parietal Lobule**	122	−14, −42, 56 −10, −48, 70	4.56 4.42	0.000077 0.00011
**Superior Parietal Lobule** **Postcentral Gyrus**	174	18, −56, 72 14, −44, 72	4.48 4.05	0.00093 0.00027
**Superior Parietal Lobule**	15	−34, −48, 44	3.93	0.00035
**SMG/AnG**	62	64, −46, 28	4.38	0.00012
**Lingual Gyrus/Calcarine Cortex**	22	18, −62, 2	4.16	0.0002
**Lingual Gyrus/Calcarine Cortex**	28	−16, −66, −2	4.12	0.00023
**Superior Occipital Gyrus**	655 *	−14, −96, 22	6.64 **	0.00000056
**Occipital Pole**	171	20, −102, 10	5.05	0.000024
**ITG/MTG**	63	−46, −12, −30	5.12	0.00002
**ITG/MTG**	72	−46, −36, −16	4.81	0.000042
**MTG**	87	−52, −52, 0	4.64	0.000063
**MTG**	18	−58, −66, −6	3.94	0.00035
**Primary Auditory Cortex** **Parietal Operculum** **Planum Temporal**	152	−48, −24, 6 −54, −38, 24 −52, −32, 16	4.66 4.42 4.09	0.00006 0.00011 0.00024

**Table 11:** Training (SndLsnHard-SndLsnEasy) sham–stim, ROI Analysis. * Significant at pFWE < 0.05 corrected for multiple comparisons within the region of interest. PMd = dorsal premotor cortex. BA44v = ventral Brodmann area 44 Broca’s area (Mask from Neubert et al., 2014 [67]).

Brain Region	Cluster Size	MNI Coordinate x, y, z	T	*p*	pFWE_corr
**Primary Auditory Cortex**	51	−48, −24, 6	4.66	0.00006	0.013 *
**PMd**	19	−26, 12, 46	4.26	0.00016	0.039 *
**BA44v**	6	−50, 22, 10	3.88	0.00041	0.030 *

## Supplementary Materials

The following supporting information can be downloaded at: https://www.mdpi.com/article/10.3390/brainsci13071024/s1, Table S1: Landing Rate Performance.
