# Supplementary material for: Simultaneous fMRI and tDCS for Enhancing Training of Flight Tasks
Source: Brain Sci. 2023 Jul 3;13(7):1024. doi: 10.3390/brainsci13071024 (PMC10377527; doi:10.3390/brainsci13071024)
Supplement: Supplementary file 1 [file brainsci-13-01024-s001.zip › brainsci-2451996-supplementary.pdf]

Supplementary Information

Simultaneous fMRI and tDCS for Enhancing Training of Flight Tasks

Jesse A. Mark [1,*], Hasan Ayaz [1,2,3,4,5,6,*] and Daniel E. Callan [7,*]

[1] School of Biomedical Engineering, Science and Health Systems, Drexel University, Philadelphia, PA 19104, USA
[2] Department of Psychological and Brain Sciences, College of Arts and Sciences, Drexel University, Philadelphia, PA 19104, USA
[3] Drexel Solutions Institute, Drexel University, Philadelphia, PA 19104, USA
[4] A.J. Drexel Autism Institute, Drexel University, Philadelphia, PA 19104, USA
[5] Department of Family and Community Health, University of Pennsylvania, Philadelphia, PA 19104, USA
[6] Center for Injury Research and Prevention, Children's Hospital of Philadelphia, Philadelphia, PA 19104, USA
[7] Brain Information Communication Research Laboratory, Advanced Telecommunications Research Institute International, Kyoto 619-0237, Japan
* Correspondence: jam673@drexel.edu (J.A.M.); ayaz@drexel.edu (H.A.); dcallan@atr.jp (D.E.C.)

Table S1. Landing rate LMM significance results for all main effects, interactions, and covariates, as well as significant post-hoc comparisons (*p<0.05, **p<0.01, ***p<0.001).

Term	Factor	F-ratio		p-value	Partial η2
**Landing Rate Main Effects (Post-Pre)**					
	tDCS	0.9	1/19.1	0.354536	0.045
	Experience	5.08	1/20.8	0.035108*	0.196
	Experience*tDCS	1.952	1/19.5	0.178011	0.091
	Runway	5.739	1/542.4	0.016928*	0.010
	Wind	1.766	1/541.3	0.184404	0.003
	Auditory	5.494	1/536.1	0.019447*	0.010
	Pre-training Performance	186.6	1/501.2	0.000000***	0.271
**Landing Rate Post-Hoc Comparisons (Post-Pre)**					
	Active stim, experience novice vs advanced	6.012	1/21.1	0.045982*	0.222
**Landing Rate Main Effects (Training)**					
	tDCS	4.147	1/19.2	0.055735	0.178
	Experience	10.49	1/21.4	0.003854***	0.329
	Experience*tDCS	4.401	1/19.7	0.049001*	0.183
	Runway	18.85	1/547	0.000017***	0.033

	Wind	2.378	1/545.4	0.123645	0.004
	Auditory	1.497	1/539.1	0.221749	0.003
	Pre-training Performance	135.8	1/447.3	0.000000***	0.233
**Landing Rate Post-Hoc Comparisons (Training)**					
	Active stim, experience novice vs advanced	12.76	1/21.8	0.003443***	0.369
	Experience novice, active stim vs sham	7.553	1/19.9	0.024894*	0.275
**Landing Rate Main Effects (Training & Post-Pre)**					
	Run	1.906	1/1102.4	0.167747	0.002
	tDCS	2.294	1/19.5	0.145927	0.105
	Experience	8.318	1/20.6	0.008989***	0.288
	Run*tDCS	1.757	1/1102.4	0.185331	0.002
	Experience*tDCS	3.278	1/19.7	0.085468**	0.143
	Runway	24.07	1/1110.4	0.000001***	0.021
	Wind	3.62	1/1108.9	0.057362	0.003
	Auditory	6.154	1/1102.5	0.013259*	0.006
	Pre-training Performance	322	1/1018.3	0.000000***	0.240
**Landing Rate Post-Hoc Comparisons (Training & Post-Pre)**					
	Active stim, experience novice vs advanced	10.02	1/20.8	0.009402***	0.325
